# Supplementary material for: Prevalence, Screening Practices and Risk Stratification for Diabetic Foot Complications in Primary Healthcare Clinics: A Cross-Sectional Study in Gauteng Province, South Africa
Source: Int J Environ Res Public Health. 2025 Nov 27;22(12):1794. doi: 10.3390/ijerph22121794 (PMC12732912; doi:10.3390/ijerph22121794)
Supplement: Supplementary file 1 [file ijerph-22-01794-s001.zip › ijerph-3861701-supplementary.pdf]

Annexure A: Questionnaire Diabetic Foot Assessment and Risk Stratification

UNIVERSITY OF JOHANNESBURG

Please cross(x) the relevant block.

Section A – Demographic Data

1. Income bracket (Hospital classification)

H0 (Free patients)	**1**
H1 (R0-R70 000p.a)	**2**
H2 (>R70 000 to R250 000p.a)	**3**
H3(> R250 000p.a)	**4**

2. Gender

Male	**1**
Female	**2**
Prefers not to say	**3**
Age in years	

3. Ethnicity

Black	**1**
Coloured	**2**
Indian or Asian	**3**
White	**4**

4. Level of education

Below grade 12	**1**
Grade 12 (Matric)	**2**
Post Grade 12	**3**
Not stated	**4**

5. Comorbidities (Mark all applicable)

Hypertension	**1**
Hypercholesterolemia	**2**
Chronic Kidney Disease	**3**
Cardiac disease	**4**
Renal disease	**5**
Retroviral disease	**6**
Other (specify)	**7**

6. Duration of diabetes

≤ 1 year	**1**
1-5 years	**2**
5-10 years	**3**
10-15 years	**4**
15-20 years	**5**
≥ 20 years	**6**

Section B - Foot assessment

Peripheral neuro assessment

Numbness	**1**
Burning	**2**
Paraesthesia	**3**
Tingling	**4**

Peripheral vascular assessment

Pulses palpable	**1**
Pulses palpable but faint	**2**
Pulses not palpable	**3**
History of intermittent claudication	**4**

Skin/Dermatological presentations

Corns	**1**
Calluses	**2**
Skin infections	**3**
Thick nails	**4**
Ingrown nails	**5**
Fissures/Cracks	**6**
Maceration between toes	**7**

Ulceration

Any active diabetic foot ulcers (if yes, indicate site below)	**Yes**	**No**
Toes		
Plantar metatarsal area		
Medial longitudinal arch		
Heel		
History of previous/healed DFUs	**Yes**	**No**

Amputation

Any history of diabetic related foot lower limb amputations (if yes, indicate site below)	**Yes**	**No**
Transmetatarsal		
Ankle		
Transtibial		
Transfemoral		

Foot Deformities

Hallux valgus	1
Prominent metatarsal heads	2
Hammer toes	3
Claw toes	4
Pes planus	5
Pes cavus	6

Diabetic foot screening history

Have you had a diabetic foot screening in the past 12 months (if yes, indicate how it was done below)	**Yes**	**No**
Healthcare professional asked about my feet		
Healthcare professional looked at my feet (if a healthcare professional did look at your feet, indicate which of the following was checked)		
Checked the skin on my feet		
Checked the nails		
Checked for foot deformities		
Checked temperature		
Checked my footwear		
Checked for sensation		
After the assessment, were you told of your risk level/category?	**Yes**	**No**

Section C: Risk Stratification per IWDGF

Risk stratification based on assessment findings as per IWDGF

**Risk Category**	**Presenting characteristics**	**Risk level**
0 (Very Low)	No LOPS or PAD	
1 (Low)	LOPS or PAD	
2 (Moderate)	LOPS and PAD or LOPS and foot deformity or PAD and foot deformity	
3 (High)	LOPS or PAD and ≥1 of foot ulcer history, lower extremity amputation, ESRD	

UNIVERSITY OF JOHANNESBURG

DEPARTMENT OF PODIATRY
RESEARCH CONSENT FORM
REC 11.0

Diabetic Foot Assessment and Risk stratification at primary healthcare level.

Please initial each box below:

☐ I confirm that I have read and understand the information letter dated Click here to enter the date, as is appears on the information sheet. for the above study. I have had the opportunity to consider the information, ask questions and have had these answered satisfactorily.

☐ I understand that my participation is voluntary and that I am free to withdraw from this study at any time without giving any reason and without any consequences to me.

☐ I agree to participate in the above research.

______________________ ________________

Signature of Participant Date

________________________ ____________________________________ ________________

Name of Researcher Signature of Researcher Date
